# Supplementary material for: Plasma-induced surface cooling
Source: Nat Commun. 2022 May 12;13:2623. doi: 10.1038/s41467-022-30170-5 (PMC9098841; doi:10.1038/s41467-022-30170-5)
Supplement: Supplementary file 1 — Supplementary Information [file 41467_2022_30170_MOESM1_ESM.pdf]

Supplementary Information: Plasma-induced surface cooling

John A. Tomko,[1,*] Michael J. Johnson,[2] David R. Boris,[3] Tzvetelina B. Petrova,[3] Scott G. Walton,[3,†] and Patrick E. Hopkins[4,1,5,‡]

[1]*Department of Mechanical and Aerospace Engineering, University of Virginia, Charlottesville, VA 22904, USA*

[2]*Syntek Technologies, Fairfax, VA 22031, USA*

[3]*Plasma Physics Division, Naval Research Laboratory, Washington DC 20375, USA*

[4]*Department of Materials Science and Engineering, University of Virginia, Charlottesville, VA 22904, USA*

[5]*Department of Physics, University of Virginia, Charlottesville, VA 22904, USA*

(Dated: April 15, 2022)

* email: jat6rs@virginia.edu

† email: scott.walton@nrl.navy.mil

‡ email: phopkins@virginia.edu

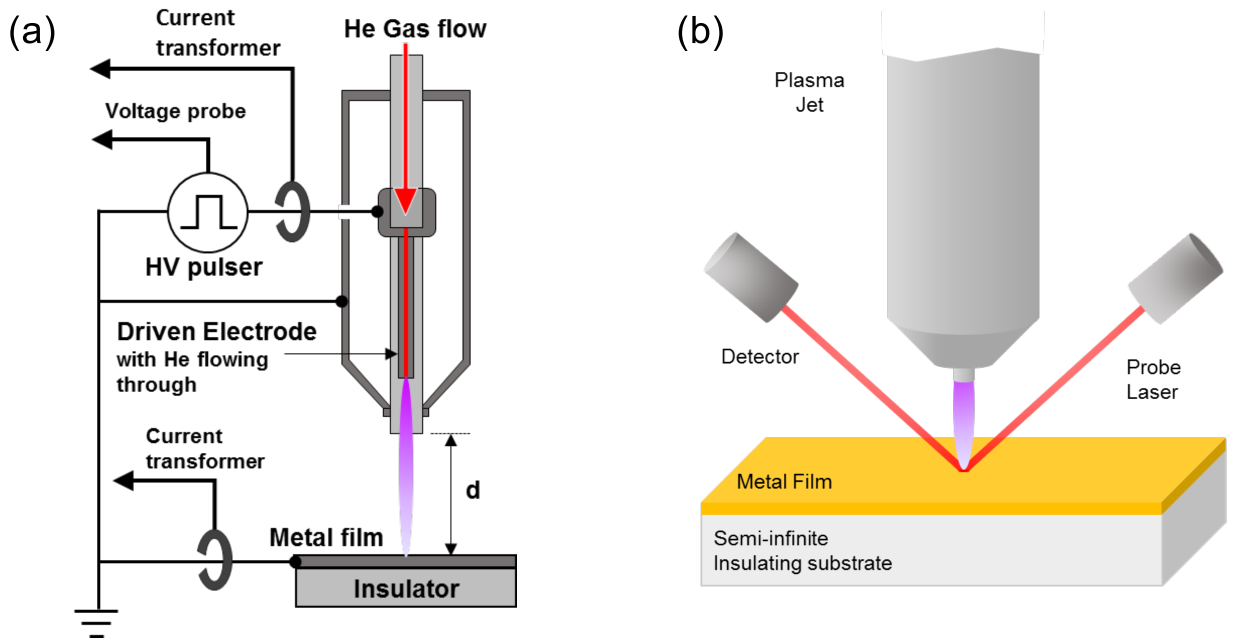

Supplementary Figure 1. Experimental configuration used in this work. a) Experimental schematic of the plasma jet, driving circuit, and electrical characterizations to determine the voltage and current applied to the driven needle electrode and current delivered to the surface[1]. b) Experimental schematic of our plasma-pump, optical-probe diagnostic tool. A pulsed atmospheric plasma jet bombards an Au surface, while a continuous wave laser monitors the thermoreflectance of the Au surface to gain insight to the plasma-surface energy transfer mechanisms.

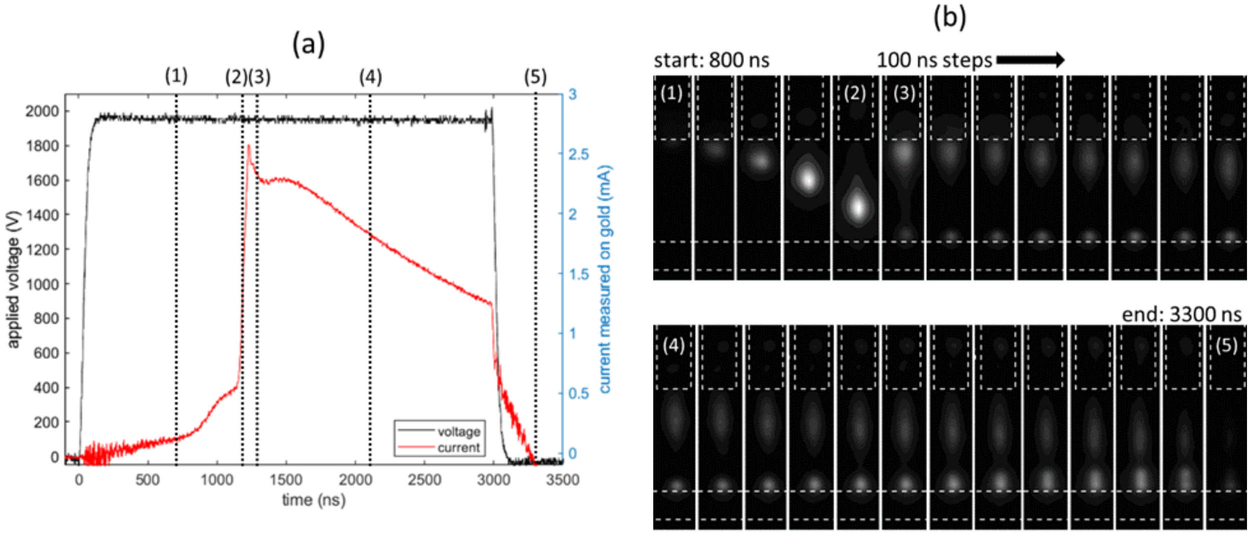

Supplementary Figure 2. Data showing the time-dependent behavior of the plasma jet interacting with a gold substrate. (a) Shows the pulsed-DC voltage applied to the plasma jet along with the current measured on the substrate and (b) shows the high speed images of the jet interacting with the substrate. The vertical lines in (a) indicate corresponding images shown in (b).

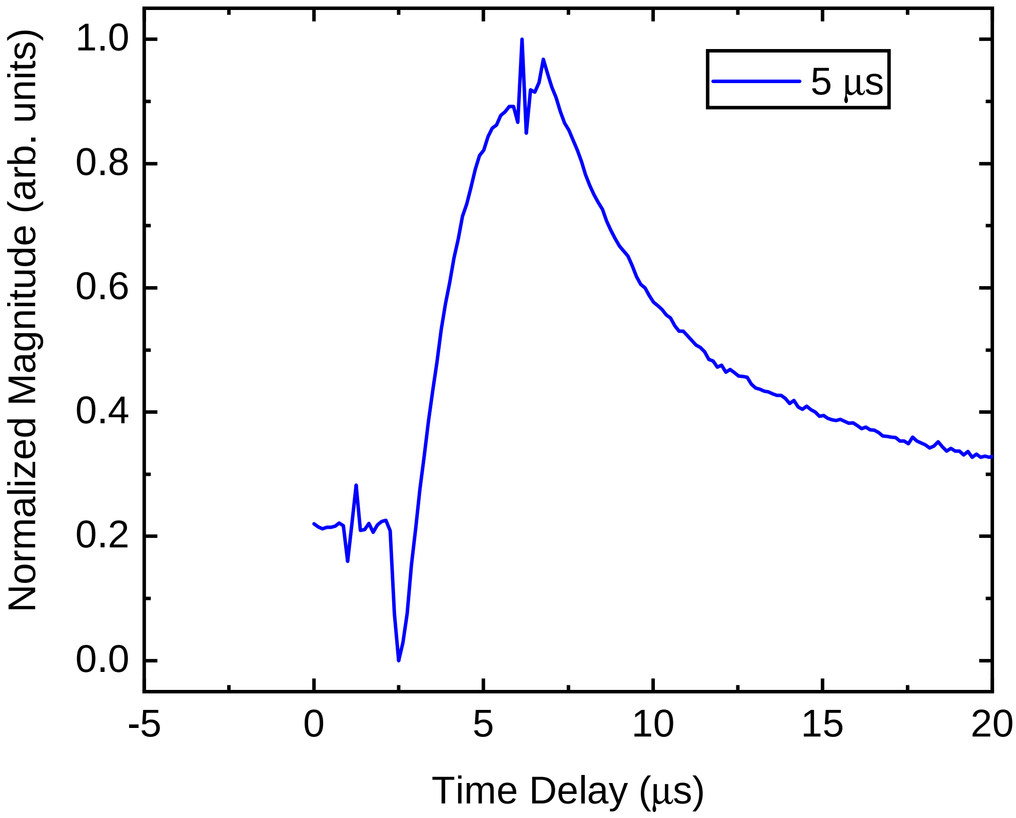

Supplementary Figure 3. Thermoreflectance measurement data at a laser probe wavelength of 405 nm. Due to the positive thermoreflectance coefficient of Au at this wavelength, these data show a decrease in reflectance during cooling and increased reflectance as the sample temperature increases.

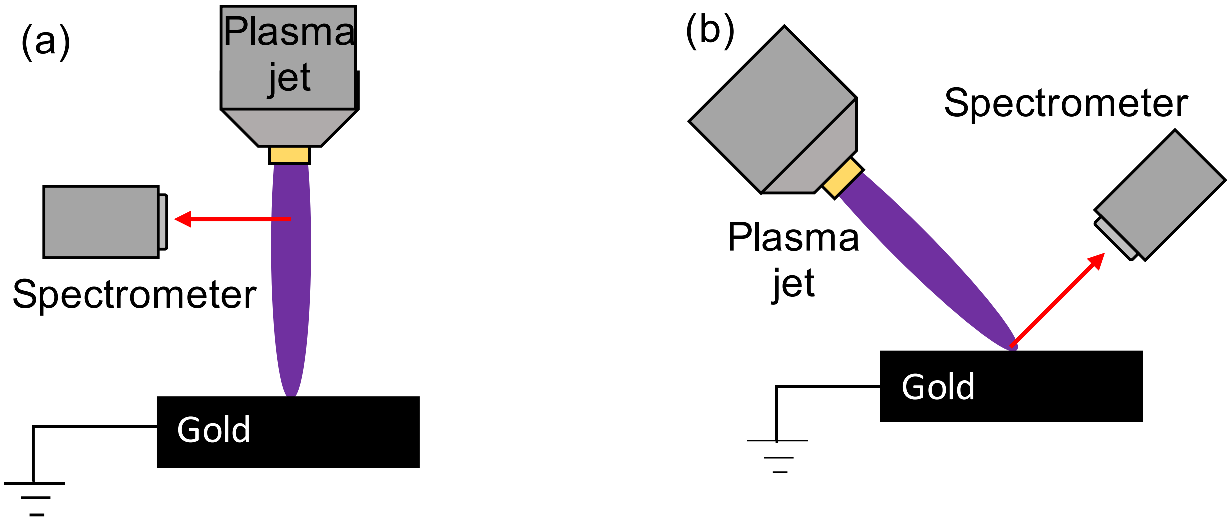

Supplementary Figure 4. Experimental configurations for measuring optical emission spectra of the plasma jet in this work. (a) Shows a typical geometry where the emission spectra is acquired through a line of sight normal to the axis of the jet. This geometry was used to acquire the spectrum in Fig. S2(b). (b) Geometry used to acquire time-dependent emission spectra as seen by the surface and shown in Fig. 3(a) of the manuscript.

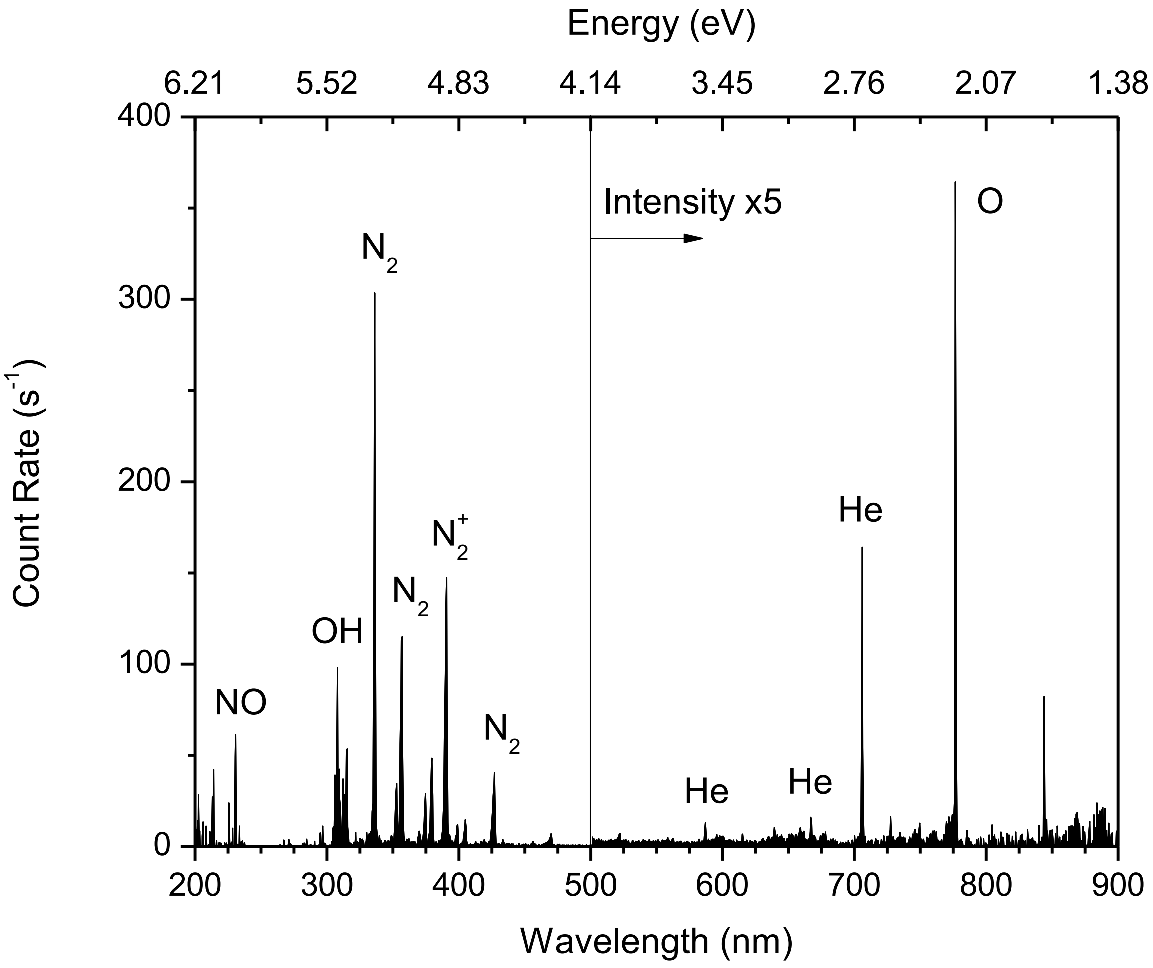

Supplementary Figure 5. Emission spectrum of a plasma jet produced in helium interacting with a gold substrate. The spectrum is acquired near the jet exit through a line of sight perpendicular to the axis of the jet. The intensity of the spectrum for wavelengths above 500 nm has been multiplied by 5.

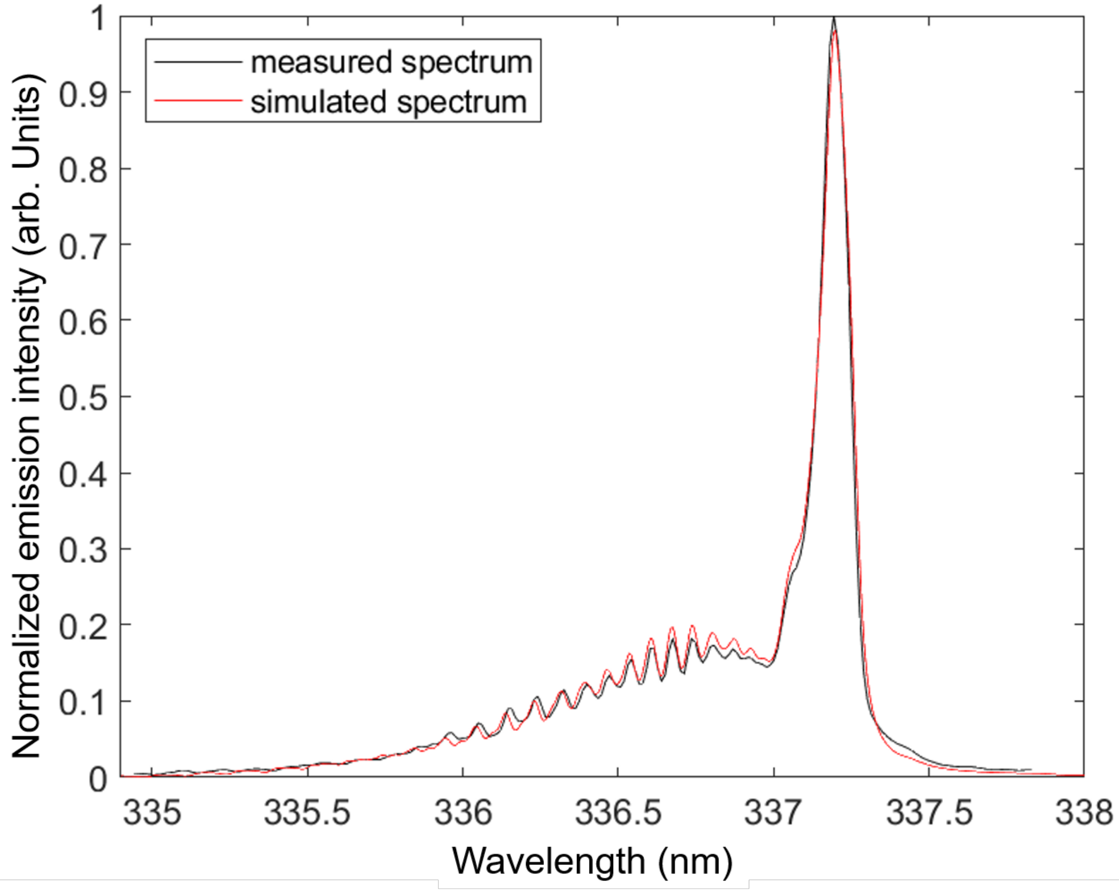

Supplementary Figure 6. N_2 emission spectrum. The measured and simulated emission spectra of the 337 nm line of the second positive system in N_2 [$N_2(C^3\prod_u \rightarrow N_2(B^3\prod_g$].

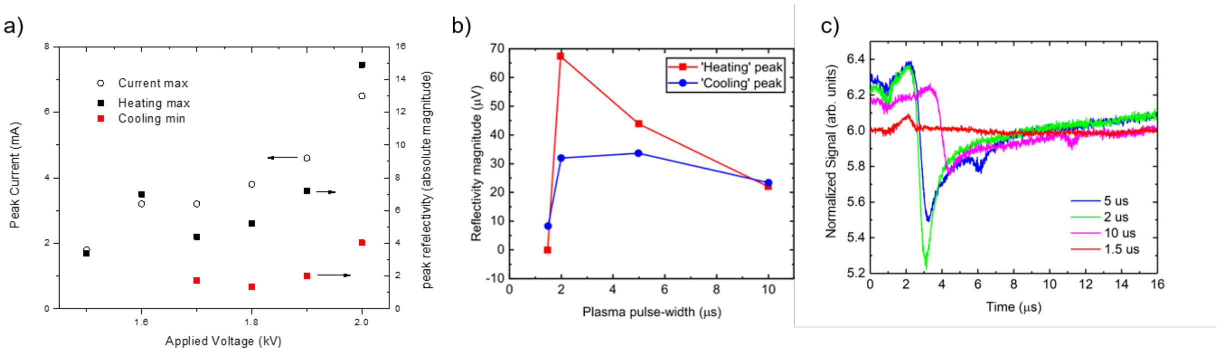

Supplementary Figure 7. Role of plasma jet parameters for modulated cooling. a) Peak of the measured surface current (black circles) and relative thermoreflectance magnitude of the heating (red circles) and cooling (blue squares) peak during plasma-Au interactions as a function of the voltage used to produce the plasma jet. b) Relative thermoreflectance magnitude of the heating (red circles) and cooling (blue squares) peak during plasma-Au interactions as a function of the pulse-width of the incident plasma jet. (c) Thermoreflectance of varying pulsed widths indicating the potential to operate in a regimes where cooling dominates the surface response (e.g., red line at a pulsed width of 1.5 μs displays negligible heating).

Supplementary Table I. Thermophysical parameters for TTM of 80 nm Au/sapphire. These parameters include the electronic heat capacity and thermal conductivity, C_e and κ_e, the lattice heat capacity and thermal conductivity, C_p and κ_p, and the electron-phonon coupling factor, G.

**Parameter**	**Au**	**Al_2O_3**
C_e (J m^{-3} K^{-1})	$68.1 * T_e$	0
C_p (MJ m^{-3} K^{-1})	2.49	3.06
κ_e (W m^{-1} K^{-1})	135	0
κ_p (W m^{-1} K^{-1})	3	35
G (10^{16} W m^{-3} K^{-1})	2.5	0

Supplementary Table II. Measured plasma temperatures. The approximate rotational and vibrational temperature taken from the 337 nm line in the second positive system for a given duration of the applied voltage.

**pulse width (μs)**	**rotational temperature (K)**	**vibrational temperature (K)**
3	301.55	1040.1
5	311.85	1004.9
7	309.04	1018.1
10	314.36	1120.3

SUPPLEMENTARY NOTE 1: ATMOSPHERIC PLASMA JET.

A schematic of our atmospheric plasma jet and experimental setup is shown in Supplementary Fig. 1(a). The details of this experimental configuration have been discussed in detail in our previous work[1]. In summary, the plasma jet consists of a hollow needle electrode centered within a ceramic tube; this tube is enclosed by a grounded, metal casing. Helium flows through the needle electrode at a constant rate (1500 sccm; measured with a mass flow controller). To drive plasma production, a high voltage pulses ($1.5-2.5$ kV) are applied to the needle for a set duration ($2-5$ μs) and frequency. For the data in this work, the operating voltage, pulse width, and frequency were 2 kV, 5 μs, and 7.79 kHz, unless noted otherwise. Our experiments are performed in laboratory air with a relative humidity of $35\% - 45\%$. The measurement setup is partially enclosed to minimize external flow from laboratory air conditioners and filtration systems that can perturb the plasma jet during laser spectroscopy measurements. The substrate in this work is an 80 nm Au film on a crystalline Al_2O_3 substrate; the metal film is located 1 cm from the exit of the jet and connected to ground. Current transformers and high voltage probes are used to measure the current to ground and power to the driven electrode.

SUPPLEMENTARY NOTE 2: PLASMA JET CHARACTERIZATION.

We employ a variety of characterization techniques to link the plasma jet operation to the surface response. The temporal behavior of the plasma jet is described in Supplementary Fig. 2 using current-voltage measurements and high-speed images acquired using an Andor 334T iStar ICCD camera[2]. The measured currents are a combination of the discharge currents associated with the plasma jet and a displacement current from the electric field induced by high voltage switching of the powered electrode. This displacement current can be quantified by comparing the measured current with and without gas flowing, since a plasma jet can not be generated in the absence of gas flow for the voltages used in this work. The surface current is thus calculated by subtracting this displacement current from the total current measured. The time delay between the increase in applied voltage and the peak in the measured current [Supplementary Fig. 2(a)] is related to the time it takes the streamer to reach the substrate and is well-correlated to the high-speed images in Supplementary Fig. 2(b). The streamer is generated at the powered electrode when the voltage is applied and pushed towards the substrate. The streamer emerges from the jet body at $\approx$900 ns

and strikes the substrate surface at $\approx$1250 ns. After contact, the current on the substrate reaches its peak and a surface discharge forms. During this phase, a measurable but decreasing current persists and photon emission (See Fig 3(a) in the main manuscript) remains similar to the streamer for the remainder of the voltage pulse. Once the voltage pulse is over, the discharge will linger, producing measurable current for another 300 ns, until it eventually dissipates. Importantly, the photon flux at the surface continues well after the current is no longer measurable.

SUPPLEMENTARY NOTE 3: TRANSIENT LASER REFLECTIVITY MEASUREMENTS.

To measure the temperature of the Au surface during plasma interactions, we perform transient thermoreflectance measurements with a laser probe, as illustrated in Supplementary Fig. 3. For this work, we focus an incident laser, with a wavelength of either 405 nm or 637 nm, to the Au surface at an incident angle of approximately 45 degrees. The reflected beam is collimated and re-focused into a silicon photodiode. The photodiode output is then sent to a pre-amplifier; this output is then measured with a lock-in amplifier (Zurich Instruments UHFLI). We use the applied voltage for the plasma production as the reference signal for the lock-in amplifier. However, rather than measuring the magnitude/phase of the reflectance, we use periodic waveform analysis (PWA; comparable to moving boxcar averaging) to obtain the transient differential reflectance that is induced by the plasma jet.

As shown in Supplementary Fig. 3, the 405 nm probe wavelength shows opposite signs in differential reflectivity for the heating/cooling phases during plasma-surface interactions compared to the transient reflectivity using a 637 nm probe (See Fig. 2 in the main manuscript). This can be attributed to the negative thermoreflectance coefficient of Au at photon energies below the interband transition threshold of the metal film ($\sim$2.5 eV) at 637 nm, and the positive thermoreflectance coefficient associated with 405 nm[3]. Despite this inversion, we see qualitatively similar dynamics between in the two measurement approaches. Specifically, a brief period of cooling precedes the rapid heating of the gold. After heating, the material then slowly relaxes.

SUPPLEMENTARY NOTE 4: TWO-TEMPERATURE MODEL AND EVAPORATIVE COOLING CALCULATIONS.

As outlined in a number of previous works, one can empirically interpret the temporal dynamics of a pump-probe reflectivity experiment via a semi-classical 'two-temperature' model (TTM)[4–6]. In conventional pump-probe experiments, a laser is used to 'pump' or excite the material, while a second laser is used to 'probe' the material response. As photons only couple their energy to electrons, on ultrafast timescales, the lattice of a metal will remain cold, while the electronic subsystem rapidly gains energy/increases in temperature when the pump laser is applied. Within hundreds of femtoseconds to a few picoseconds the electrons impart their energy to the lattice, with this time scale being determined by the temperature differential between the electronic and phononic subsystems as well as an electron-phonon coupling factor. Ultimately, this energy transfer follows two governing equations,

$$C_e(T_e)\frac{\delta T_e}{\delta t} = \nabla \cdot (\kappa_e \nabla T_e) - G(T_e - T_p) + S(t) \tag{1}$$

$$C_p(T_p)\frac{\delta T_p}{\delta t} = \nabla \cdot (\kappa_p \nabla T_p) + G(T_e - T_p) \tag{2}$$

where C_e and C_p are the heat capacities of the electrons and phonons, respectively, T_e and T_p are the temperatures of the electrons and phonons, respectively, and κ_e and κ_p are the thermal conductivities of the electrons and phonons, respectively. G represents the electron-phonon coupling factor and $S(t)$ is the heating source, which includes the relevant parameters of the ultrafast pump pulse. In this work, we perform $multi-layer$ TTM calculations, using literature values for the relevant Au parameters[4–6], as well as experimentally measured/calculated values for the Al_2O_3 substrate; these parameters are listed in Supplementary Table 1. Note, there is no electronic contributions to heat transfer for Al_2O_3, and thus the relevant parameters are not listed. And since the plasma jet replaces a laser as the energy source in our 'plasma-pump, laser probe' experiments, we directly insert the measured surface current trace as the primary heating source, $S(t)$, in Eq. 1.

To understand how cooling via either an electronic or atomic channel affects the temporal dynamics, we introduce a second 'source' term to either Eq. 1 or Eq. 2, respectively. To mimic electronic ejection, we simply implement a Gaussian cooling event that reduces the surface temperature of the electronic subsystem, with a peak temperature decay of 1 K. This is mimicked for the case of adsorbate removal, where the cooling term is inserted to Eq. 2. In both cases, the

best-fit occurs with a time-scale of cooling of 800 ns. However, we note that due to the time scales investigated in this work, our thermoreflectance measurement is insensitive to whether the cooling occurs through electronic or atomic processes; a temporal resolution on the order of 100s of femtosecond to a picosecond would be necessary to directly interrogate and separate these processes with laser-based spectroscopy techniques.

In the case of material removal for evaporative cooling, we can directly calculate the theoretical energy loss (i.e., change in temperature) associated with the process of atomic desorption from the target surface based on the specific heat of species within our probed measurement volume. Based on the spot size ($\approx 55\mu$m) and skin depth ($\approx$ 15 nm) of our laser in Au, the probed volume is $\approx$150 μm^3. Pairing this volume with the volumetric heat capacity of Au, $\approx$2.49 MJ m^{-3} K^{-1}, a local 1 K temperature reduction would require 0.37 nJ to be removed from the probed volume. At room temperature, a reasonable approximation to the average thermal energy of each particle in a solid is simply $3k_BT$, or $\approx 1.24\times10^{-20}$ J. With these values in mind, it would require 3×10^{10} atoms to be removed from the probed volume; this number of atoms is equivalent to approximately a single monolayer of material removed homogeneously across the area of the laser probe. If we consider that the cooling is induced by the removal of adsorbed water, which has a heat capacity of $\approx$4.2 MJ m^{-3} K^{-1}, a similar temperature decrease requires sub-monolayer thicknesses, or non-uniform distributions, of water to be removed from the surface. When considering a similar calculation for electron ejection, the energy removed from the probe volume would remain 0.37 nJ.

We note that the above estimations assume an instantaneous removal of energy at the peak temperature reduction for an estimate on the order of magnitude required for our observations. For cooling over the course of 800 ns, heat conduction from the now-hotter surrounding substrate will lead to an under-estimation of the total energy required for a 1 K reduction in the peak temperature. In other words, we would anticipate larger volumes of material or adsorbates to be removed. Indeed, our best-fit results for one-dimensional TTM calculations, which provide good agreement with our experimental data, suggest that a significantly larger amount of energy is removed from the system; over the course of 800 ns, approximately 25 nJ of energy would have to be removed. Returning to the above calculations of material/adsorbate removal, this energy would require very large volumes of Au removal, further supporting the posit of adsorbate evaporation from the Au surface. However, we note that this calculation is simply an estimate and does not account for the 3-dimensional nature of heat conduction and plasma interactions (recall: the plasma source is significantly larger than the laser probe).

SUPPLEMENTARY NOTE 5: PHOTOEMISSION MEASUREMENTS.

To understand the relative flux of species at the surface, optical emission spectroscopy was employed to measure both time-averaged broadband emission as well as time-resolved emission intensity of select lines. The geometry for these measurements is shown in Supplementary Fig. 4. An Ocean Optics NIR spectrometer was used to measure time-averaged emission spectrum from the jet using the geometry shown in Supplementary Fig. 4(a). Those results are shown in Supplementary Fig. 5 and indicate a wide range of photons - extending from UV to IR - are produced by the plasma jet. VUV and EUV photons ($<$ 200 nm) has also been observed in He jets[7], but are not observable in our experiments due to bandwidth limitations and strong absorption in air. The spectrum also indicates that, despite being produced in a helium flow, a wide variety of air-related products are produced in the plasma due to the downstream mixing of helium and air. While useful for species identification, the spectrum lacks any spatial or temporal information. To best understand the reflectivity results, the time-resolved flux of photons at the surface must be measured. To do this, we employed the geometry shown in Supplementary Fig. 4(b), which provides a line-of-sight along the axis of the jet and thus the ability to measure emission incident to the surface from ignition through the afterglow. Select emission lines were monitored using a a scanning monochromator (2035 McPherson) with a photomultiplier tube (McPherson model number 654) connected to a multi-channel scaler (Stanford Research Systems model SR430 multichannel scaler) to accumulate the data. Those results are shown in Fig. 3 of the main article and compared to the time-dependent voltage and current measurements.

While it is difficult to make a non-intrusive measurement of the gas temperature within an atmospheric pressure plasma, the rotational temperature of N_2 can be used as an approximation[8]. To measure the rotational temperature, the emission from the second positive system was measured over the entire voltage pulse at the plasma jet-substrate interface and is shown in Supplementary Fig. 6. This spectrum was compared to a simulated spectrum generated that followed a Boltzmann distribution generated using the software package 'MassiveOES'[9,10]. The rotational and vibrational temperature that produced the simulated spectrum closest to the measured spectrum was determined and is shown in Supplementary Table 2. Regardless of the length of the voltage pulse, the rotational temperature never gets significantly higher than room temperature, as it reaches 314 K over the longest tested pulse width. This approximation is based on a time-averaged measurement of the N_2 rotational lines during the entire pulse duration. Accordingly, this estimate should

be considered as average gas temperature during the time the plasma is active.

SUPPLEMENTARY NOTE 6: RESULTS VS. PLASMA JET PARAMETER.

We consider the relative heating and cooling contributions with varying plasma-jet parameters such as applied voltage and it's duration or pulse width. As the applied voltage of the electrodes is increased, we observe a corresponding increase in the measured surface current, as well as an increase in the peak temperature of our thermoreflectance measurements [Supplementary Fig. 7(a)]. When the applied voltage pulse width is varied, a significant change in peak temperature is seen, while the minimum temperature achieved during cooling is not significantly changed [Supplementary Fig. 7(b)]. When above a critical voltage pulse width, increasing the pulse width further has little impact on the cooling peak, while the heating peak changes noticeably [see Supplementary Fig. 7(b)]. Shown Supplementary Fig. 7(c) is a comparison of reflectivity curves at select pulse widths. The results in Supplementary Fig. 7(c) and Supplementary Fig. 7(c), suggest it is possible to operate in a regime where the surface heating is decreased to a point where the surface experiences a net reduction in temperature.

SUPPLEMENTARY REFERENCES

[1] S. G. Walton, B. M. Foley, J. Tomko, D. R. Boris, E. D. Gillman, S. C. Hernández, A. Giri, Tz B. Petrova, and P. E. Hopkins, "Plasma-surface interactions in atmospheric pressure plasmas: In situ measurements of electron heating in materials," Journal of Applied Physics **124**, 43301 (2018).

[2] Michael J. Johnson, David R. Boris, Tzvetelina B. Petrova, and Scott G. Walton, "Spatio-temporal characterization of a pulsed DC atmospheric pressure plasma jet interacting with substrates," Journal of Physics D: Applied Physics **54**, 085202 (2021).

[3] R. B. Wilson, Brent A. Apgar, Lane W. Martin, and David G. Cahill, "Thermoreflectance of metal transducers for optical pump-probe studies of thermal properties," Optics Express **20**, 28829 (2012).

[4] J. K. Chen, D. Y. Tzou, and J. E. Beraun, "A semiclassical two-temperature model for ultrafast laser heating," International Journal of Heat and Mass Transfer **49**, 307–316 (2006).

[5] A. Block, M. Liebel, R. Yu, M. Spector, Y. Sivan, F. J. García De Abajo, and N. F. Van Hulst, "Tracking ultrafast hot-electron diffusion in space and time by ultrafast thermomodulation microscopy," Science Advances **5**, eaav8965 (2019), arXiv:1809.10591.

[6] Elizabeth L. Radue, John A. Tomko, Ashutosh Giri, Jeffrey L. Braun, Xin Zhou, Oleg V. Prezhdo, Evan L. Runnerstrom, Jon Paul Maria, and Patrick E. Hopkins, "Hot Electron Thermoreflectance Coefficient of Gold during Electron-Phonon Nonequilibrium," ACS Photonics **5**, 4880–4887 (2018).

[7] Judith Golda, Beatrix Biskup, Vincent Layes, Tristan Winzer, and Jan Benedikt, "Vacuum ultraviolet spectroscopy of cold atmospheric plasma jets," Plasma Processes and Polymers **17**, e1900216 (2020).

[8] P.J. Bruggeman, N. Sadeghi, D.C. Schram, and V. Linss, "Gas Temperature Determination from Rotational Lines in Non-Equilibrium Plasmas: A Review," Plasma Sources Sci. Technol. **23**, 023001 (2014).

[9] J. Vorac, P. Synek, V. Prochazka, and T. Hoder, "State-by-state emission spectra fitting for non-equilibrium plasmas: OH spectra of surface barrier discharge at argon/water interface," J. Phys. D. Appl. Phys. **50**, 294002 (2017).

[10] J. Vorac, P. Synek, L. Potocnakova, J. Hnilica, and V. Kudrle, "Batch processing of overlapping molecular spectra as a tool for spatio-temporal diagnostics of power modulated microwave plasma jet," Plasma Sources Sci. Technol. **26**, 025010 (2017).
